# Heteroexpression of *Aspergillus nidulans* *laeA* in Marine-Derived Fungi Triggers Upregulation of Secondary Metabolite Biosynthetic Genes

**DOI:** 10.3390/md18120652

**Published:** 2020-12-18

**Authors:** Ishrat Khan, Wan-Lin Xie, Yu-Chao Yu, Huan Sheng, Yan Xu, Jia-Qi Wang, Sanjit Chandra Debnath, Jin-Zhong Xu, Dao-Qiong Zheng, Wan-Jing Ding, Pin-Mei Wang

**Affiliations:** Ocean College, Zhejiang University, Zhoushan 316021, China; ishratkhan@zju.edu.cn (I.K.); xiewanlin@zju.edu.cn (W.-L.X.); yuyuchao@zju.edu.cn (Y.-C.Y.); shenghuan@zju.edu.cn (H.S.); xuyan875@zju.edu.cn (Y.X.); wangjiaqihelen@zju.edu.cn (J.-Q.W.); sanjit.c.debnath@zju.edu.cn (S.C.D.); xujinzhong@zju.edu.cn (J.-Z.X.); zhengdaoqiong@zju.edu.cn (D.-Q.Z.); wading@zju.edu.cn (W.-J.D.)

**Keywords:** *Aspergillus*, heteroexpression, LaeA, fungi, secondary metabolites

## Abstract

Fungi are a prospective resource of bioactive compounds, but conventional methods of drug discovery are not effective enough to fully explore their metabolic potential. This study aimed to develop an easily attainable method to elicit the metabolic potential of fungi using *Aspergillus nidulans laeA* as a transcription regulation tool. In this study, functional analysis of *Aspergillus nidulans laeA* (AnLaeA) and *Aspergillus* sp. Z5 *laeA* (Az5LaeA) was done in the fungus *Aspergillus* sp. Z5. Heterologous AnLaeA-and native Az5LaeA-overexpression exhibited similar phenotypic effects and caused an increase in production of a bioactive compound diorcinol in *Aspergillus* sp. Z5, which proved the conserved function of this global regulator. In particular, heteroexpression of AnLaeA showed a significant impact on the expression of velvet complex genes, diorcinol synthesis-related genes, and different transcription factors (TFs). Moreover, heteroexpression of AnLaeA influenced the whole genome gene expression of *Aspergillus* sp. Z5 and triggered the upregulation of many genes. Overall, these findings suggest that heteroexpression of AnLaeA in fungi serves as a simple and easy method to explore their metabolic potential. In relation to this, AnLaeA was overexpressed in the fungus *Penicillium* sp. LC1-4, which resulted in increased production of quinolactacin A.

## 1. Introduction

Marine fungi are a prolific source of discovering new drugs with tremendous bioactivities [1,2,3,4,5]. Many studies revealed the noteworthiness of these microorganisms as a prospective resource of pharmaceutically important antifungal, antibacterial, antiviral, anti-inflammatory, anticancer, enzyme inhibitor, and antitumor compounds [6]. Industrial investments in natural product research have decreased because conventional methods for the discovery of natural products cannot fulfil the increasing need of bioactive compounds in the healthcare system [7]. Many genetic manipulation methodologies, for instance, transcriptional regulation, epigenetic regulation, ribosome engineering, and heterologous expression, have been tested to analyze biosynthetic processes at different gene regulation levels in different fungi [8,9,10], but there is still a need to choose an easily attainable approach that could also work in those unstudied fungi whose genomes are not explored.

**Figure 1:**
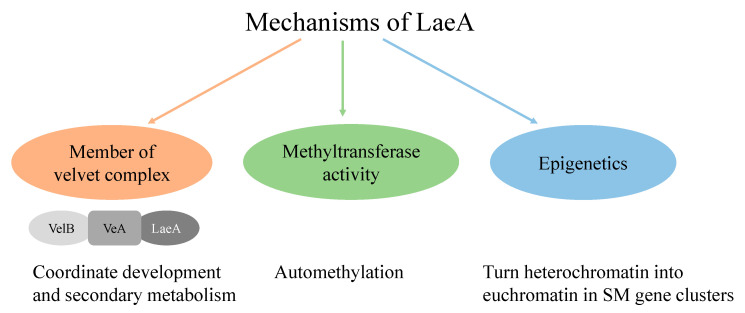
Schematic representation of LaeA mechanisms [21,22,23].

Global regulators regulate the expression of secondary metabolite (SM) biosynthetic gene clusters [7]. The discovery of global regulator LaeA in *Aspergillus* spp. by Bok and Keller has revolutionized the understanding of fungal secondary metabolism [11]. The dynamic roles of LaeA have been studied in many aspects of fungal biology, such as in asexual and sexual differentiation, in phenotype changes including pigmentation and sporulation [12,13], reducing [14] or increasing secondary metabolite production [11,15,16], and activation of cryptic gene clusters [13,17]. Numerous studies based on model *Aspergillus nidulans* LaeA have been carried out during the past several years, which proved its mechanisms (Figure 1) and regulatory role in secondary metabolism [11,18,19,20]. In the present study, we hypothesized that if the function of LaeA is conserved among filamentous fungi, then *A. nidulans laeA* can be used as a transcription regulation tool to upregulate biosynthetic gene clusters (BGCs) in fungi. The proposed method may exclude the need for genome sequencing techniques, and it could be a quick way to explore the metabolic potential of fungi. To demonstrate the conserved role of LaeA, functional analysis of *A. nidulans laeA* (AnLaeA) and *Aspergillus* sp. Z5 *laeA* (Az5LaeA) was done in the host *Aspergillus* sp. Z5. It was observed that overexpression of both regulatory genes has similar effects on the phenotype and SM production of strain Z5. Additionally, comparative transcriptome analysis showed a substantial effect of AnLaeA overexpression on the whole genome gene expression of *Aspergillus* sp. Z5. Hence, this study suggests that AnLaeA can be directly used as a transcription regulation tool to elicit the secondary metabolism of fungi. Further studies are in progress to overexpress AnLaeA in fungi belonging to different genera.

## 2. Results

### 2.1. Bioinformatics Analysis of Az5LaeA and AnLaeA

**Figure 2:**
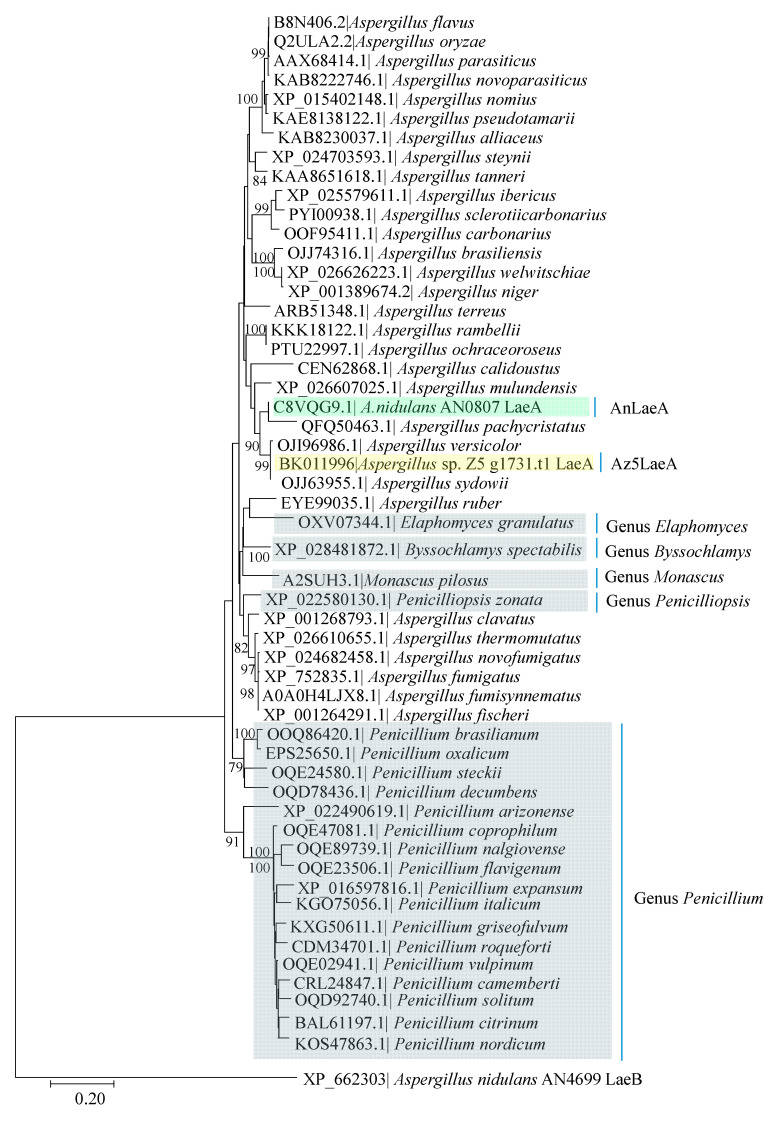
Neighbor-joining (NJ) tree based on LaeA protein sequences, showing the relationship between *Aspergillus nidulans laeA* (AnLaeA) and its closest orthologs. Numbers at nodes indicate bootstrap values (≥ 70%) based on 1000 replicates. Each branch is labelled with the species name and its corresponding LaeA protein accession number. Different genera belonging to the phylum *Ascomycota*, are boxed in grey, while the unboxed species belong to the genus *Aspergillus*. Functionally analyzed AnLaeA is boxed in green, and *Aspergillus* sp. Z5 *laeA* (Az5LaeA) is boxed in yellow. *A. nidulans* AN4699 LaeB was included in the tree as an outgroup.

The open reading frame (ORF) of Az5LaeA encodes a 352 amino acid peptide. BLASTP analysis showed that Az5LaeA (LaeA of *Aspergillus* sp. Z5, BK011996) shares a high degree of similarity with its orthologs of different genera (Table S1). Multiple sequence alignment of *Aspergillus* sp. Z5 LaeA showed conserved domain (Figure S1). Protein sequence analysis by InterProScan revealed that it is a S-adenosyl-L-methionine-dependent methyltransferase (SAM MTase). Phylogenetic analysis of well-characterized AnLaeA (LaeA of *A. nidulans*, XP_658411) supported that LaeA is conserved in a broad range of species belonging to the genus *Aspergillus* and other genera of the phylum *Ascomycota* (Figure 2). The AnLaeA exhibited high degree similarity (86.07%) with the Az5LaeA. With an idea to use AnLaeA as a transcription regulation tool, we first attempted to prove the conserved function of this global regulator, therefore functional analysis of both *laeA* genes was done in the marine-derived fungus *Aspergillus* sp. Z5.

### 2.2. Overexpression of laeA Genes

*LaeA*-overexpression plasmids were constructed by placing AnLaeA or Az5LaeA coding regions under the control of *A. nidulans gpdA* promoter and *trpC* terminator with G418-resistance marker. The plasmid replicator AMA1 sequence was inserted into *laeA*-overexpression plasmids for autonomous plasmid replication. AMA1 (MATEs or mobile *Aspergillus* transformation enhancers) is derived from *A. nidulans*. This sequence does not encode any extended polypeptide. AMA1 sequence improves the transformation frequency up to 2000 times in contrast to the traditional integrated plasmids [24,25]. Az5LaeA and AnLaeA were overexpressed in *Aspergillus* sp. Z5 for function analysis. To validate the proposed method, AnLaeA was further overexpressed in the fungus *Penicillium* sp. LC1-4.

### 2.3. Phenotype and Scanning Electron Microscope Analysis

**Figure 3:**
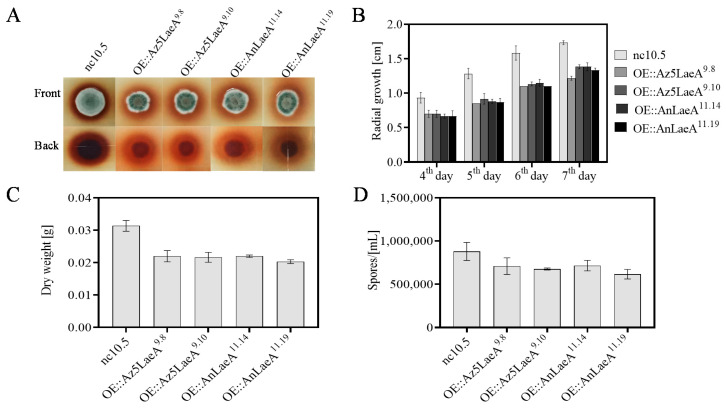
Phenotype analysis of *Aspergillus* sp. Z5 transformants. (**A**) Radial growth at 5th day of growth on solid glucose minimal medium (GMM) + G418, (**B**) quantification of radial growth on solid GMM + G418, (**C**) dry weight of transformants culture in liquid glucose minimal medium (LMM) + G418, and (**D**) spore production on solid GMM + G418. All phenotype analyses were done at 30 °C in the dark. All results are represented as the average ± SE of triplicate samples. For radial growth and dry weight, *p*-value between control nc10.5 and transformants is < 0.05. For spores production, *p*-value between control nc10.5 and transformant OE::Az5LaeA^9.10^/OE::AnLaeA^11.19^ is < 0.05.

**Figure 4:**
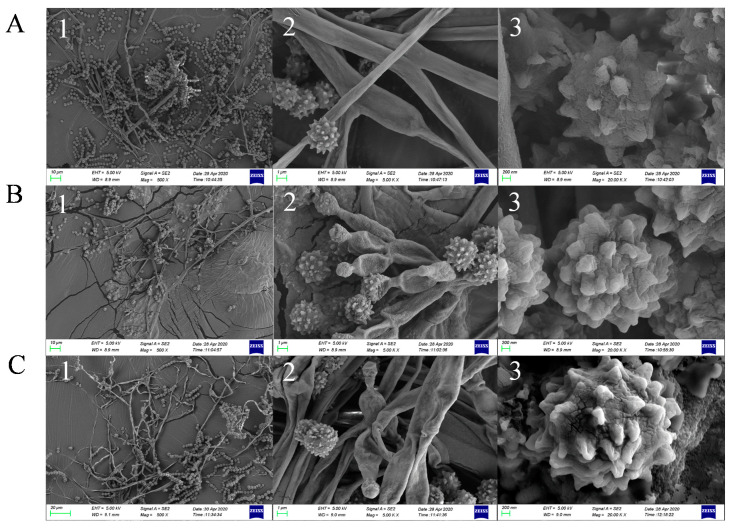
Scanning electron micrographs. (**A**) nc10.5, (**B**) OE::Az5LaeA^9.8^, (**C**) OE::AnLaeA^11.19^. 1: The distribution and abundance of spores at 500 ×; 2: Mycelial morphology at 5K ×; 3: Spore morphology at 20K ×.

The OE::AnLaeA and OE::Az5LaeA transformants of *Aspergillus* sp. Z5 showed significant phenotype differences, i.e., reduced spore production, low dry weight, and decreased radial growth compare to the negative control transformant nc10.5 (Figure 3). Scanning electron microscopy (SEM) analysis also displayed differences in the distribution of spores and in mycelial and spore morphology. *LaeA*-overexpression transformants exhibited the occurrence of less spores, and more sulcate colonies with more pronounced spore roughening (Figure 4).

### 2.4. Secondary Metabolites Analysis

**Figure 5:**
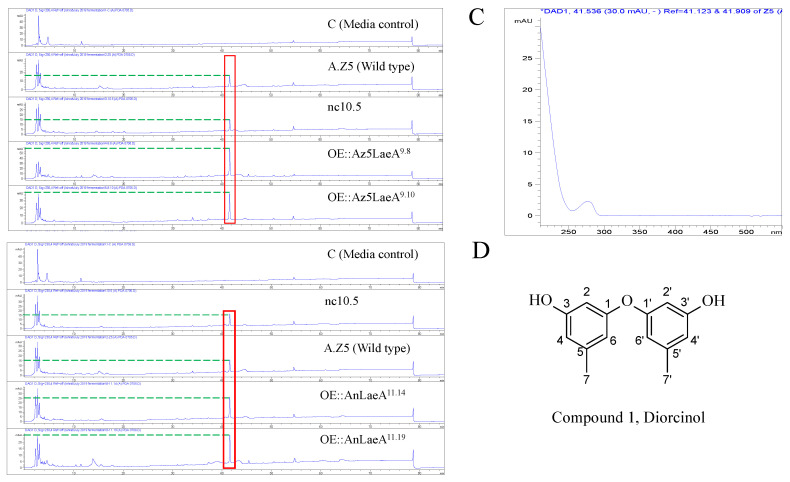
High-performance liquid chromatography (HPLC) analysis of potato dextrose broth (PDB) crude extracts of *Aspergillus* sp. Z5 transformants at UV 230 nm. (**A**) OE::Az5LaeA transformants; (**B**) OE::AnLaeA transformants; (**C**) HPLC-UV-DAD spectrum of the upregulated peak of the upregulated compound 1; (**D**) structure of compound 1, diorcinol.

**Figure 6:**
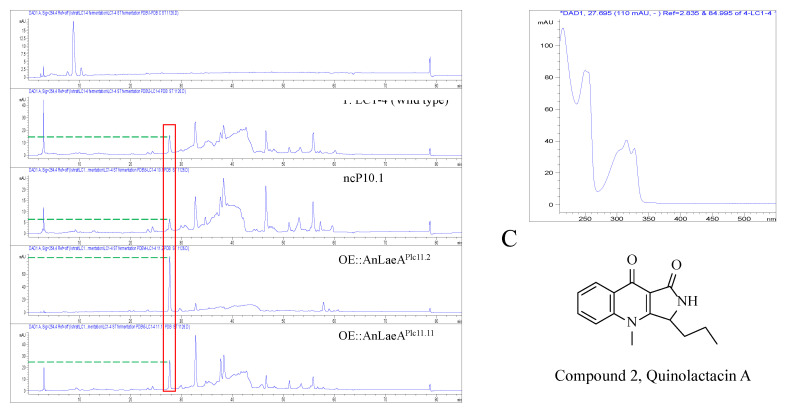
HPLC analysis of PDB crude extracts of *Penicillium* sp. LC1-4 transformants at UV 254 nm. (**A**) OE::AnLaeA transformants; (**B**) HPLC-UV-DAD spectrum of the upregulated peak of the upregulated compound; (**C**) structure of compound 2, quinolactacin A.

High-performance liquid chromatography (HPLC) analysis revealed the high production of a compound by OE::AnLaeA and OE::Az5LaeA transformants compared to the negative control transformant nc10.5 and wild type *Aspergillus* sp. Z5 (Figure 5). The increased production of compound 1 was obvious by the high peak area in *laeA*-overexpression transformants (Table 1). The native Az5LaeA-overexpression delivered more compound production compared to the heterologous AnLaeA-overexpression, but both *laeA* genes caused the upregulation of a similar compound that established the conserved function of this regulator. The structure of upregulated compound 1 in *Aspergillus* sp. Z5 was determined by comparing ^1^H and ^13^C NMR data and mass spectroscopy results with the literature [26,27]. Compound 1 was identified as 3, 3′-dihydroxy-5, 5′-dimethyldiphenyl ether, also known as diorcinol (Figures S2 and S3). The molecular weight of the compound was determined by [M + H] ion peak at *m/z* 229.0863 in HR-ESI-MS spectrum (Figure S4). Compound 1 possesses cytotoxic activity against the HCT116 human colon cancer cell line with IC_50_ 10µg. This is probably the first reported cytotoxic activity for this compound against the HCT116 human colon cancer cell line. After functional analysis in *Aspergillus* sp. Z5, AnLaeA was heteroexpressed in a fungus LC1-4 belonging to the genus *Penicillium*. Heteroexpression of AnLaeA in *Penicillium* sp. LC1-4 resulted in up to 4-fold high production of a previously identified compound 2 in transformant OE::AnLaeA ^Plc11.11^ (Figure 6) (Table 2). The secondary metabolite results of this study provide a basis for engineering *Aspergillus* and *Penicillium* species to get a high yield of pharmaceutically important compounds such as diorcinol and quinolactacin.

### 2.5. Transcriptome Analysis

To find out the genome-wide effects of AnLaeA overexpression, transcriptome analysis of nc10.5 and OE::AnLaeA^11.19^ was done at 60 h in potato dextrose broth (PDB) media. A total of 98,179,444 raw reads were generated from strain nc10.5 and OE::AnLaeA^11.19^ that accounts for about ~15 GB of paired-end sequencing data. The raw data is available on the NCBI Sequence Read Archive (SRA) under the accession number PRJNA649367. The pre-processing of raw reads was done for removal of adaptor sequences and low-quality reads. Ninety-eight million high-quality reads were retained after Cutadapt software (NBIS, Uppsala University, Uppsala, Sweden) [28] filtering (Table S2). The results show that the quality and data volumes of transcriptome sequencing were relatively high that meet the requirements for subsequent data assembly and processing. For the assembly process, reads from each strain were pooled together to reconstruct all the transcripts. Using trinity with default parameters [29], a total of 33,747 assembled transcripts were generated with a mean size of 2131 bp. 18,923 unigenes of mean length 1368 bp were obtained for a total of 25,898,836 bases (Table S3). The range in unigene length was from 201 bp to 26,466 bp, and the unigene length distribution is shown in Figure S5.

### 2.6. Functional Annotation and Differential Gene Expression of Unigenes

The assembled unigenes were annotated for sequence similarity search and comparison using BLASTX against a non-redundant protein database at NCBI with an E-value cut off 1.00 × 10^−5^. The BLASTX results showed 7662 annotated unigenes, and 11,261 remained non-annotated unigenes out of 18,923 assembled unigenes. The unigenes showed top-hit species similarity with *A. sydowii* (74.94%), *A. versicolor* (7.33%), *A. calidoustus* (0.97%), *A. mulundensis* (0.69%), and others (16.05%) (Figure S6). The annotations of unigenes against proteins in the Pfam database showed 3792 significant hits. Moreover, 7662, 5313, 3910, 3808, and 2117 unigenes were annotated against NR, STRING, SWISS-PROT, GO, and KEGG databases, respectively (Table S4). Gene Ontology (GO) classification was used to classify assembled unigenes based on annotations. 3809 unigenes were assigned to one or more GO terms, which were allocated to three major categories and 66 subcategories. In biological function category, 11,886 unigenes were assigned to 30 classes. This category includes proteins highly involved in the cellular process (2634 unigenes), metabolic process (2336 unigenes), biological regulation (982 unigenes), and regulation of biological process (839 unigenes). In cellular components category, 13,401 unigenes were assigned to 19 classes, which include proteins highly encoded in the cell (2804 unigenes), cell part (2786 unigenes), organelle (2131 unigenes), and organelle part (1299 unigenes). The molecular functions were grouped into 17 classes, with the most assignments to binding (2209 unigenes), catalytic activity (2077 unigenes), and transporter activity (373 unigenes) (Figure S7). The KEGG annotation results for unigenes showed that 2117 (11.19%) unigenes were mapped to 349 KEGG pathways. The unigenes could be classified into six branches according to the associated KEGG pathways, as shown in Figure S8. Based on the calculation and screening results, 6428 genes were determined to be differentially expressed between transformants nc10.5 and OE::AnLaeA^11.19^, among which 2679 genes were upregulated and 3749 genes were downregulated. Of these differentially expressed genes (DEGs), 445 genes with adjusted P values (*Padj*), less than 0.05 and log_2_ (fold change) values greater than 1 were assigned as significantly differentially expressed, with which 147 genes were upregulated and 298 genes were downregulated in OE::AnLaeA^11.19^ (Figure S9). Forty-three putative secondary metabolites (SM) gene clusters were predicted by antiSMASH 5.0 based on the genome sequence of *Aspergillus* sp. Z5, including 16 NRPSs, 9 PKSs, 8 terpenes, 6 hybrids, and 4 indoles. 479 unigenes were identified by sequence alignment against 417 SM genes of strain *Aspergillus* sp. Z5. Of the 417 putative SM genes, 176 genes were differentially expressed between n10.5 and OE::AnLaeA^11.19^. Among these differentially expressed SM genes, 70 genes were upregulated in OE::AnLaeA^11.19^, including 6 SM cluster backbone genes (1 PKS, 2 NRPSs, and 3 Terpenes).

### 2.7. In Vitro Validation of Specific Genes by qPCR

**Figure 7:**
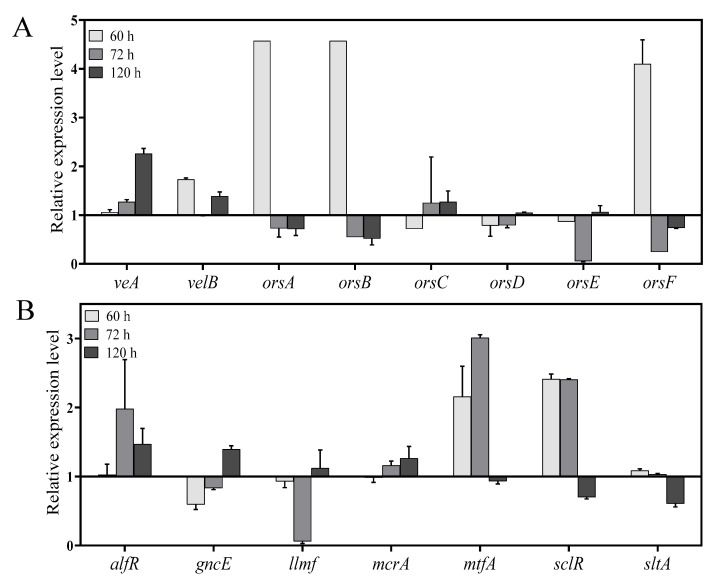
(**A**) Relative expression level of velvet complex genes (*veA* and *velB*) and diorcinol-synthesis related genes (*orsA*, *orsB*, *orsC*, *orsD*, *orsE,* and *orsF*). (**B**) Relative expression level of different transcription factors (TF). Relative expression is determined in transformant OE::AnLaeA^11.19^ based on qPCR at different time points. The expression level in negative control transformant nc10.5 is 1, which is denoted by baseline to show differential expression in transformant OE::AnLaeA^11.19^. Bars above the baseline show upregulation, while bars below the baseline show downregulation of genes.

**Figure 8:**
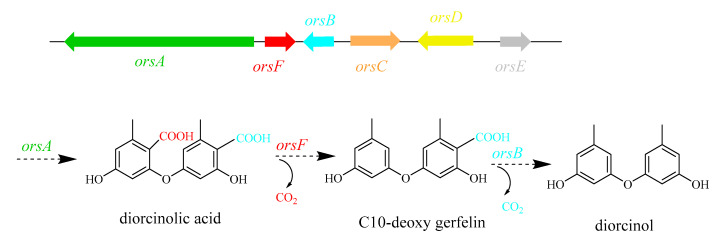
Schematic representation of biosynthetic pathway of diorcinol. Modified from that of Feng et al. [27].

The relative expression level of velvet complex genes, diorcinol-synthesis related genes and different transcription factors (TFs) involved in the regulation of growth and secondary metabolism was checked at 60 h, 72 h, and 120 h. Both velvet complex genes *veA* and *velB* showed relatively high expression at 60 h and 120 h (Figure 7A). According to the secondary metabolite results, OE::AnLaeA^11.19^ exhibited high production of diorcinol. Feng and co-workers determined three enzymes from *A. nidulans* and defined their roles in the biosynthesis of diorcinol [27]. The biosynthetic mechanism of diorcinol includes a rare polyketide synthase (PKS), AN7909 (*orsA*) [30], which is responsible for production of diorcinolic acid, while an uncharacterized enzyme, AN7910 (*orsF*) [31], and a putative amidohydrolase, AN7911 (*orsB*) [30], are responsible for catalyzing regioselective decarboxylation of diorcinolic acid to produce diorcinol (Figure 8). According to their findings, three genes, AN7912 (*orsC*), AN7913 (*orsD*), and AN7914 [32], of the *Ors* gene cluster are evidently not involved in the biosynthesis of diorcinol [27]. qPCR results of this study displayed differential expressions of diorcinol-synthesis related genes at different time points. Gene *orsA* (g2173.t1, ortholog of AN7909), *orsB* (g2171.t1, ortholog of AN7911), and *orsF* (g2172.t1, ortholog of AN7910) showed high expression in transformant OE::AnLaeA^11.19^ at 60 h (Figure 7A). These are the key genes involved in the biosynthesis of diorcinol. Gene expression analysis supported the high production of diorcinol in OE::AnLaeA^11.19^ under heteroexpression of AnLaeA (Figure 7A). LaeA has a broad impact on growth and secondary metabolism of fungi [11,33,34]. By comparing the Web Gene Ontology Annotation Plot (WEGO), enrichment of annotated unigenes, and AnLaeA upregulated genes, 26 GO categories were found enriched with AnLaeA upregulated genes (Figure S10). The main categories related to secondary metabolism with AnLaeA upregulated genes include the ‘‘metabolic process’’ category (GO: 0008152, 33 genes, 1.41%), ‘‘translation regulator activity’’ category (GO: 0045182, 1 gene, 2.85%), and ‘‘transcription regulator activity’’ category (GO: 0030528, 3 genes, 2.45%). In the transcription regulation category, 2 genes involved in transcription regulation (g1727.t1, g742.t1) and CCAAT-binding transcription factor *hapB* [35] (g5897.t1) were found upregulated in transformant OE::AnLaeA^11.19^ at 60 h. To further evaluate this category, the relative expression level of some of the key TFs was determined by qPCR (Figure 7B). *AflR* (sterigmatocystin Zn_2_CyS_6_ transcription factor) was upregulated in AnLaeA overexpression transformant OE::AnLaeA^11.19^ at 72 h and 120 h. The other TFs, *mtfA* (C_2_H_2_ transcription factor involved in the regulation of secondary metabolism) [36] and *sclR* (involved in sclerotial production) [37], showed significant upregulation at 60 h and 72 h, while *sltA* (C_2_H_2_ transcription factor involved in morphogenesis and biosynthesis of sterigmatocystin) [38] was not significantly upregulated at these time points, but all three TFs were found downregulated at 120 h. McrA is a master regulator of secondary metabolism in the genus *Aspergillus* [39]. *McrA* was positively regulated by AnLaeA at 72 h and 120 h. AnLaeA overexpression also influenced the expression of *llmf* (negative regulator of development and sterigmatocystin) [40]. *Llmf* was downregulated at 60 h and 72 h, but showed upregulation at 120 h. *GcnE* (H3K9 acetyltransferase of SAGA complex) showed high expression at 120 h (Figure 7B).

## 3. Discussion

Filamentous fungi are a prospective resource of bioactive compounds. Many strategies have been introduced to explore the metabolic potential of these natural assets [8,9,10], but most of them are difficult to handle and require genome sequencing of the strain. Global regulators can improve the whole fungal natural product diversity [7]. Therefore, this study presents an easily attainable molecular method based on utilizing the global regulator of secondary metabolism LaeA from *A. nidulans* as a transcription regulation tool in filamentous fungi. This method involves heteroexpression of AnLaeA by an autonomously replicating plasmid in fungi. It is a very simple method and easy to perform. The significance of this method includes: (1) It does not require genome sequencing of host fungi; (2) this method is applicable to fungi belonging to the family *Trichocomaceae*; (3) if fungal strains are sensitive to the same drug marker, then the same plasmid can be transformed directly to elicit their secondary metabolism without the need of plasmid construction.

In this study, functional analysis of AnLaeA and Az5LaeA was conducted in the marine-derived fungus *Aspergillus* sp. Z5. AnLaeA-and Az5LaeA-overexpression caused characteristic changes in the phenotype and delivered increased production of a bioactive compound diorcinol in *Aspergillus* sp. Z5. Our phenotype and secondary metabolite results are consistent with other *laeA*-overexpression reports, such as overexpression of *laeA* ortholog caused reduced sporulation in *A. fumisynnematus* and altered the mycelial morphology of *Monascus purpureus* [15,16]. In the previous studies, native *laeA*-overexpression has also been reported with increased production of compounds including lavostatin, monacolin K, penicillin, and cyclopiazonic acid [11,15,16]. We found that heteroexpression of AnLaeA and native Az5LaeA exhibited similar phenotypic and metabolic effects, which proved that the function of this global regulator is conserved.

Emphasizing the regulatory effect of AnLaeA, overexpression of AnLaeA showed significant impact on the expression of velvet complex genes, diorcinol-synthesis related genes, and different TFs, which justified the results of phenotype analysis and secondary metabolite analysis of this study. This study also supports that LaeA positively regulates the *Ors* gene cluster. Moreover, the comparative transcriptome analysis revealed that heteroexpression of AnLaeA can influence the whole genome gene expression of host fungi due to the conserved function. The proposed method was further validated in fungus belonging to the genus *Penicillium*. Heteroexpression of AnLaeA caused increased production of quinolactacin A in *Penicillium* sp. LC1-4, which was the main compound of this strain (Figure 6). Besides, we also tried to knockout Az5LaeA to activate some BGCs, but knockout was unsuccessful after several trials. According to our experience, knockout of global regulators may be challenging in some strains compared to the overexpression strategy.

In conclusion, heteroexpression of well-studied AnLaeA in a broad range of fungi serves as an efficient method to explore their metabolic potential. This study suggests that AnLaeA can be used as a transcription regulation tool in fungi.

## 4. Materials and Methods

### 4.1. Strains, Media, and Culture Conditions

*Aspergillus* sp. Z5 was isolated from the gut of marine isopod *Ligia oceanica* in our previous study [41], and *Penicillium* sp. LC1-4 was isolated from the intestine of a marine conch. *A. nidulans* RDIT 2.3 was obtained from Dr. Nancy Keller [11]. Strain *Aspergillus* sp. Z5 and *Penicillium* sp. LC1-4 were identified by an internal transcribed spacer (ITS) sequence, and the sequence data were deposited to GenBank under the accession number MN636770 and MT672583, respectively. Fungal strains were cultured and maintained on glucose minimal medium (GMM) agar plates [42] at 30 °C for 4 days. Spore suspensions were prepared in 0.1% Tween 80 and preserved as 25% *v/v* glycerol stocks at −80 °C. Liquid GMM supplemented with 0.5% yeast extract was used to harvest mycelia. GMM added with 1.2M D-sorbitol and supplemented with 100 µg/mL G418 (Solarbio, Shanghai, China) was used for screening of resistant transformants. For compound production, strains were cultured in PDB (200 g/L potatoes, 34 g/L ocean salts, and 20 g/L glucose) at 28 °C, 160 rpm for 10 days. Fungal strains used in this study are listed in Table 3.

### 4.2. Gene Cloning and Bioinformatics Analysis

The genomic sequence of AnLaeA was obtained from the Aspergillus genome database (http://aspergillusgenome.org/). The 1255 bp open reading frame (ORF) of AnLaeA is comprised of two exons and one intron. Exons were amplified from genomic DNA of *A. nidulans* RDIT2.3 and joined together by modified double joint PCR method [43], followed by ligation into pMD^TM^ 19-T Vector (TaKaRa, Beijing, China) to generate pISH1 plasmid. The Az5LaeA was found as AnLaeA orthologue. It is comprised of 1249 bp ORF and shares 86.07% identity with AnLaeA. Az5LaeA ORF was cloned from genomic DNA of *Aspergillus* sp. Z5 using specific primers (Table S5). The Az5LaeA was analyzed by an online BLAST search at the National Center for Biotechnology Information (NCBI) web site (https://blast.ncbi.nlm.nih.gov/Blast.cgi) [44]. Multiple sequence alignment of Az5LaeA coding sequence with other LaeA correlatives from diverse genera was done by ClustalW2 (EMBL-EBL, Hinxton, Cambridgeshire, UK) (https://www.ebi.ac.uk/Tools/msa/clustalw2/) [45]. The theoretical isoelectric point (pI) and molecular weight of Az5LaeA protein was determined by ExPASy (SIB, Switzerland) (https://web.expasy.org/compute_pi/) [46]. The conserved domain of Az5LaeA protein was scanned by the InterProScan (EMBL-EBL, Hinxton, Cambridgeshire, UK) (http://www.ebi.ac.uk/interpro/search/sequence/) [47]. *LaeA* gene sequences of other correlatives from diverse genera were retrieved from NCBI to deduce the phylogenetic affiliation of well characterized AnLaeA. Phylogenetic analysis was carried out using MEGA7 software (https://megasoftware.net/) [48].

### 4.3. Molecular Genetic Manipulations

The backbone of overexpression plasmids was amplified from pC-G418-YR plasmid (http://www.addgene.org/61767/), that was comprised of resistant kanamycin (*kanR*) gene for *Escherichia coli*, *URA3* gene for yeast, and resistant G418 (*neoR/kanR*) gene for fungi as selectable markers with Ori and 2µ origin of replication for *E.coli* and yeast, respectively. To construct autonomously replicating plasmids, AMA1 was obtained from ANEp2 plasmid (http://fgsc.net/) by restriction endonuclease (RE) *Not* I digestion. A constitutive *gpdA* promoter and *trpC* terminator were cloned from *A. nidulans* RDIT 2.3 using specific primers (Table S5). The ORF of Az5LaeA and the coding region of AnLaeA were placed between promoter and terminator to generate *laeA*-overexpression plasmids pISH9 and pISH11, respectively. A negative control plasmid pISH10 without *laeA* gene was also constructed to check the null effect plasmid elements. Plasmid maps are shown in Figure S11. All the PCR amplified and RE digested fragments were gel purified and transformed into *Saccharomyces cerevisiae* strain BJ5464 to generate circular plasmids by yeast homologous recombination [49]. Plasmids were transformed into competent *E. coli* strain DH5α for propagation and extracted to transform into fungi [13]. Plasmids details are shown in Table S6.

### 4.4. Fungal Transformation

*Aspergillus* sp. Z5 was transformed with negative, Az5LaeA-, and AnLaeA-overexpression plasmids. The strain was first grown on a GMM plate at 30 °C for 4 days. *Aspergillus* sp. Z5 germlings were prepared by culturing 5–6 mL 10^7^ sp/mL in LMM at 30 °C, 200 rpm for about 12–14 h. Germlings were harvested by centrifugation at 5000 rpm, 4 °C, and washed twice with sterilized ddH_2_O. The harvested germlings were then digested to generate protoplasts by enzymatic preparation in 10 mL osmotic media (259.8 g/L MgSO_4_, 10 mM NaPhosphate buffer, pH 5.8 with 1M Na_2_HPO_4_) as a buffer with following enzymes in respective concentrations: 0.01g/mL lysozyme (Sangon Biotech, Shanghai, China), 0.01 g/mL snailase (BBI Life Sciences, Shanghai, China), 0.01g/mL lysing enzyme (SIGMA, St. Louis, MO, USA), and 0.005 g/mL yatalase (TaKaRa, Beijing, China) at 30 °C, 80 rpm for 6 h. The solution after enzyme digestion was layered by slow release of 10 mL trapping buffer (109.3 g/L D-Sorbitol, 0.1 M Tris-HCI, pH 7.0) and centrifuge at 5000 rpm, 4 °C for 20 min. The clear white protoplast layer formed in the interface was collected and washed with STC buffer (218.6 g/L D-Sorbitol, 0.47 g/L CaC1_2_ and 10 mM Tris-HCI, pH 7.5) at 5000 rpm, 4 °C for 10 min. For transformation, 100 μL protoplasts 10^7^ p/mL was mixed with ≤ 75 μL (15–20 μg) transforming DNA (dissolved in STC buffer) and incubated on ice for 50 min. 1.25 mL polyethylene glycol (PEG) solution (60 g/L PEG, 50 mM CaC1_2_, 50 mM Tris-HCl, pH 7.5) was added into the transforming mixture, mixed well, and incubated for 20 min at room temperature. The final volume of the mixture was made up to 10 mL with STC buffer. Plating was done using SMM + G418 100 µg/mL agar media. 1 mL transforming mixture was poured per plate and overlaid with 3.5 mL 0.8% SMM agar top media. Plates were incubated at 30 °C in the dark for 2 days. pISH10 and pISH11 were transformed into *Penicillium* sp. LC1-4 by following the same transformation procedure, but with a different enzymic preparation, i.e., 0.0055g/mL lysing enzyme (SIGMA, St. Louis, MO, USA) and 0.0025g/mL yatalase (TaKaRa, Beijing, China) in 10 mL osmotic medium. Secondary screening of all transformants was done on GMM + G418 (100 µg/mL). Resistant transformants were cultured into LMM + G418 200 µg/mL for 2 days. Those transformants which were able to grow in the presence of the drug were subjected to further analysis. Extraction of genomic DNA was performed as defined by Green [50]. Confirmation of positive transformants was done by diagnostic PCR using gene specific primers (Table S5). Those transformants that contained the *gpdA-laeA* and *neoR/kanR* gene sequence were considered as positive *laeA*-overexpression transformants. While confirmation of negative control transformants was done by checking *gpdA-trpC* and *neoR/kanR* gene sequences.

### 4.5. Phenotype Analysis and Scanning Electron Microscopy

Phenotype analyses of *Aspergillus* sp. Z5 transformants were performed. In phenotype analysis, transformants were examined for their radial growth, dry weight, and spore production. To examine the radial growth, 5 µL (10^4^ spores in total) of each transformant was point-inoculated onto GMM + G418 (100 µg/mL) agar plates. Colony diameter was measured from day 4 to day 7 of growth at 30 °C in the dark. Spore production was determined on GMM + G418 (100 µg/mL) agar plates. Briefly, 10 mL 0.8% molten agar top layer that contained 10^7^ spores in total for each transformant was overlaid on GMM + G418 (100 µg/mL) plates. Plates were incubated at 30 °C for 7 days in the dark. An agar plug of 1.5 cm in diameter was removed with the help of a sterilized cork borer and homogenized in 2 mL 0.1% tween 80 to make a uniform spore suspension. Spores were counted using a Neubauer chamber. Dry weight was determined by inoculating 10^7^ spores of each transformant in 4 mL LMM + G418 (100 µg/mL) and cultured at 30 °C for 4 days in the dark. Mycelia were collected and dried in a freeze dryer at −55 °C, 5 Pa for 3 h. Dried mycelia were kept at −80 °C over night, and the weight was determined. All experiments were performed in triplicate. Mycelial and spore morphology of transformant nc10.5, OE::Az5LaeA^9.8^ and OE::AnLaeA^11.19^ were observed by using scanning electron microscopy (SEM) (ZEISS Sigma, Jena, Germany). Transformants were point inoculated onto GMM + G418 (100 µg/mL) agar plates and cultured at 30 °C for 5 days in the dark. Samples were prepared as follows; the conductive double-sided adhesive carbon tape was stuck on small metal grids and then the grids were gently punched onto the growth of transformants to take the impression. Samples were observed at 500 ×, 5K×, and 20K× magnification to analyze the arrangement of spore chain, mycelial, and spore morphology.

### 4.6. Secondary Metabolite Analysis, Purification, and Identification of Compound

Secondary metabolite analysis was initially done on a small scale. The wild type of *Aspergillus* sp. Z5 was activated on GMM agar plates, while the negative control, OE::Az5LaeA, and OE::AnLaeA transformants of *Aspergillus* sp. Z5 were activated on GMM + G418 (200 mg/mL) agar plates. 150 µL of each strain’s spore suspension (10^6^ sp/mL) was spread over PDA plates and incubated at 28 °C for 7 days in the dark. Uniform size agar pieces of each grown strain were inoculated into 250 mL PDB media. Every strain was cultured in triplicate. Strain *Aspergillus* sp. Z5, nc10.5, OE::Az5LaeA^9.8^, OE::Az5LaeA^9.10^, OE::AnLaeA^11.14^, and OE::AnLaeA^11.19^ were incubated at 28 °C, 160 rpm for 10 days. Fermentation was done in the dark. For shaking fermentation, extraction was done by adding 250 mL methanol in each flask, and then flasks were sonicated for 55 min. The obtained metabolite extracts were dissolved in methanol. All samples were analyzed by HPLC (Agilent 1260, Santa Clara, CA, USA) using a C18 (Cosmosil; 5 µm, 4.6 × 250 mm) column with MeOH/H_2_O (10-100% v/v) at 1 mL/min for 60 min. Upregulation of the compound was found in all *Aspergillus* sp. Z5 transformants. OE::Az5LaeA transformant OE::Az5LaeA^9.8^ was fermented in 13 L PDB medium under same culturing conditions to get the upregulated compound 1. The fermentation broth (13 L) was filtered and extracted three times with an equal volume of EtOAc to obtain 845.6 mg crude extract. The crude extract was fractionated by silica gel column chromatography and eluted progressively using a gradient of petroleum ether(P)-EtOAc(E) (20:1−1:1) to obtain 5 fractions (Frs. 1–5) based on TLC analysis. All fractions were analyzed by HPLC with a C18 (Cosmosil; 5 µm, 4.6 × 250 mm) column at 230 nm. Fr.5 (63.5 mg) was found containing targeted compound 1, therefore Fr.5 was proceeded for semi-preparative HPLC (Agilent C18 column: 10 μm, 10 × 250 mm) with ACN/H_2_O (40/60, v/v) at 1 mL/min for 40 min to obtain compound 1 (25.53 mg). ^1^H and ^13^C NMR Spectrum of compound 1 was determined in CD3OD. The molecular weight of the compound was determined by liquid chromatography mass spectrometry LC-MS (Agilent 6230, Santa Clara, CA, USA). The cytotoxic activity of the compound was evaluated against the HCT116 human colon cancer cell line [51]. The HCT116 cells were from the Cell Bank of China Science Academy (Shanghai, China). The small-scale fermentation for *Pencillium* sp. LC1-4 and its transformants was done similar to *Aspergillus* sp. Z5. Fermentation was done at 28 °C under static condition for 1 month. Upregulation of compound 2 was found in all *Pencillium* sp. LC1-4 transformants. For compound 2, large-scale fermentation (50L PDB) was done for wild type of *Pencillium* sp. LC1-4 in a previous work (unpublished work). The purification and identification of compound 2 was done following the same procedure as described for compound 1 of *Aspergillus* sp. Z5.

### 4.7. Transcriptome Analysis

To evaluate the genome wide effect of AnLaeA-overexpression, a comparative transcriptome analysis was done between transformant nc10.1 and OE::AnLaeA^11.19^. Transformants were cultured in PDB at 30 °C, 200 rpm for 60 h. High quality RNA samples underwent RNA-sequencing. Before the library construction, poly(A) mRNAs were isolated with magnetic Oligo-dT beads (Invitrogen, Carlsbad, CA, USA), and then cDNA libraries were constructed and sequenced by SeeGene Biotech Co., Ltd., Taizhou, China. Briefly, 5 μg of total RNA for each transformant was used for the construction of libraries using TruSeq RNA sample prep Kit (Illumina, San Diego, CA, USA), according to the manufacturer’s protocol. The constructed DNA template was enriched by PCR amplification (15 cycles). Amplicons were collected and purified by Certified Low Range Ultra Agarose (Bio-Rad, Hercules, CA, USA) gel electrophoresis. Before sequencing, the DNA libraries were quantified using TBS-380 Mini-Fluorometer with PicoGreen reagent (Invitrogen, Carlsbad, CA, USA). Clone clusters were generated on Illumina cBot, using Truseq PE Cluster Kit v3-cBot-HS, and high-throughput sequencing was performed on the Illumina Hiseq4000 Truseq SBS Kit v3-HS (300 cycles). The read quality of the RNA-Seq libraries was evaluated using FastQC (Illumina Inc., San Diego, CA, USA) (http://www.bioinformatics.babraham.ac.uk/projects/fastqc/) [52]. The raw reads were cleaned by removing adapter sequences, unknown bases from 5′end, low-quality sequences with over 10% unknown bases ‘N’, reads with a quality score lower than 20 and reads with the length lower than 25 bp by Cutadapt v1.16 software (NBIS, Uppsala University, Uppsala, Sweden) [28] available at https://cutadapt.readthedocs.io/en/stable/. Reads number, error ratio, Q20, Q30, and GC-content were calculated. All of the downstream analyses were based on high quality clean reads. De novo assembly of the high-quality reads into full-length transcripts was operated by Trinity v2.6.6 (Broad Institute of MIT and Harvard, Cambridge, MA, USA) [29], which consists of inchworm, chrysalis, and butterfly modules. The process begins with inchworm, which generates full-length transcripts from raw reads based on default k-mer values. The chrysalis clusters the contigs generated by inchworm and prepares de Bruijn graph for each cluster. Finally, the butterfly module processes individual graphs, reporting full-length transcripts for alternatively spliced isoforms. The functional annotation of the de novo assembled sequences was performed using a BLASTX search (the E-value cut off was 1.00 × 10^−5^) in several protein databases, including the NR (Non-Redundant) database (https://www.ncbi.nlm.nih.gov/), the SWISS-PROT database (https://www.uniprot.org/), KEGG (Kyoto Encyclopedia of Genes and Genome) database (https://www.genome.jp/kegg/), and STRING (Search Tool for the Retrieval of Interacting Genes) (https://string-db.org/), based on sequence similarity. Unigenes were annotated according to top hits against known sequences. Differential expression analysis, gene ontology (GO) enrichment analysis, and pathway enrichment analysis were conducted as described before [53]. Genes with adjusted *p* values (*Padj*) less than 0.05 and log_2_ (fold change) values greater than 1 were assigned as differentially expressed. GO terms and pathways with *p* values less than 0.05 were considered significantly enriched.

### 4.8. In Vitro Validation of Specific Genes by qPCR Analysis

Quantitative PCR (qPCR) was performed to verify the differential expression levels between transformant nc10.1 and OE::AnLaeA^11.19^ for diorcinol-synthesis related genes, velvet complex genes, and different TFs involved in the regulation of growth and secondary metabolism (Table S7). Transformants nc10.1 and OE::AnLaeA^11.19^ were cultured in PDB media at 30 °C and 200 rpm. Mycelia were collected at 60 h, 72 h, and 120 h. RNA was isolated from mycelia using TRIzol™ reagent (Invitrogen, Shanghai, China), according to the manufacturer’s instructions. Each strain RNA sample underwent cDNA synthesis. Briefly, RNA samples were treated with RQ1 RNase-Free DNase Kit (Promega, Madison, WI, USA) to remove genomic DNA. Reverse transcription was done to synthesize cDNA using PrimeScript One Step RT-PCR Kit (TaKaRa, Beijing, China). qPCR was performed using LightCycler 480 (Roche, Rotkreuz, Switzerland) with TB Green^®^ Fast qPCR Mix (TaKaRa, Beijing, China), according to the manufacturer’s instructions. Transformant’s cDNA samples were used as templates for qPCR. Specific primers to each gene are listed in Table S5. The qPCR thermal cycling conditions for all reactions were as follows: A single cycle of pre amplification at 94 °C for 30 s, 40 cycles of amplification at 94 °C for 5 s, and 60 °C for 10 s, a single cycle of melting at 95 °C for 5 s, 60 °C for 1 min and 95 °C continuous, and the final single cycle hold at 50 °C for 30 s. All reactions were performed in triplicate, and results were expressed as relative expression levels to the *Aspergillus* sp. Z5 actin gene. The Ct (cycle threshold) values obtained were used as the original data to calculate the relative expression level of different genes to the actin gene by the 2^-ΔΔCt^ method [54,55].

## 5. Conclusions

In conclusion, the function of global regulator LaeA is conserved in filamentous fungi, as heterologous AnLaeA and native Az5LaeA overexpression showed similar effects on phenotype and secondary metabolism of the host strain. Comparative transcriptome analysis revealed that heteroexpression of AnLaeA can influence the whole genome gene expression and induce the secondary metabolism of host fungi. Therefore, AnLaeA can be used as a transcription regulation tool in other fungi.

## Figures and Tables

**Table 1:** HPLC information of upregulated compound peak in wild type, negative control, Az5LaeA-and AnLaeA-overexpression transformants of *Aspergillus* sp. Z5. All results are represented as average ± SE of triplicate samples. For peak area, *p*-value between wild type *Aspergillus* sp. Z5/control nc10.5 and transformants is < 0.05.

Strains	UV (nm)	Retention Time (min)	Width (min)	Height (mAU)	Area (mAU*s) (Average ± SE)
*Aspergillus* sp. Z5	230	41.58	0.17	12.5	152.8 ± 8.58
nc10.5	230	41.61	0.17	11.7	151 ± 20.80
OE::Az5LaeA^9.8^	230	41.59	0.17	52.9	512.3 ± 73.53
OE::Az5LaeA^9.10^	230	41.57	0.18	35.3	418.9 ± 22.33
OE::AnLaeA^11.14^	230	41.57	0.17	22.2	239.7 ± 15.94
OE::AnLaeA^11.19^	230	41.60	0.17	21.8	340.7 ± 45.76

**Table 2:** HPLC information of upregulated compound peak in wild type, negative control, and OE::AnLaeA transformants of *Penicillium* sp. LC1-4.

Strains	UV (nm)	Retention Time (min)	Width (min)	Height (mAU)	Area (mAU*s)
*Penicillium* sp. LC1-4	254	27.66	0.28	14	282.4
ncP10.1	254	27.68	0.30	5.1	109.4
OE::AnLaeA^Plc11.2^	254	27.72	0.24	85.5	1376.1
OE::AnLaeA^Plc11.11^	254	27.72	0.25	24.8	414.7

**Table 3:** List of fungal strains used in this study.

Strain Name	Parental Strain	Genotype	Source
*Aspergillus nidulans* RDIT2.3	*Aspergillus nidulans*	*veA1*	[11]
*Aspergillus* sp. Z5	Wild type	Wild type	[41]
nc10.5	*Aspergillus* sp. Z5	*ama1 gpdA trpC neoR/kanR*	This study
OE::Az5LaeA^9.8^	*Aspergillus* sp. Z5	*ama1 gpdA::Az5LaeA::trpC neoR/kanR*	This study
OE::Az5LaeA^9.10^	*Aspergillus* sp. Z5	*ama1 gpdA::Az5LaeA::trpC neoR/kanR*	This study
OE::AnLaeA^11.14^	*Aspergillus* sp. Z5	*ama1 gpdA::AnLaeA::trpC neoR/kanR*	This study
OE::AnLaeA^11.19^	*Aspergillus* sp. Z5	*ama1 gpdA::AnLaeA::trpC neoR/kanR*	This study
*Penicillium* sp. LC1-4	Wild type	Wild type	
ncP10.1	*Penicillium* sp. LC1-4	*ama1 gpdA trpC neoR/kanR*	This study
OE::AnLaeA^Plc11.2^	*Penicillium* sp. LC1-4	*ama1 gpdA::AnLaeA::trpC neoR/kanR*	This study
OE::AnLaeA ^Plc11.11^	*Penicillium* sp. LC1-4	*ama1 gpdA::AnLaeA::trpC neoR/kanR*	This study

## Supplementary Materials

The following are available online at https://www.mdpi.com/1660-3397/18/12/652/s1, Figure S1: Multiple sequence alignment of Az5LaeA with its orthologs by ClustalX2.1, Figure S2: ^1^H NMR Spectrum of compound 1 in CD3OD, Figure S3: ^13^C NMR Spectrum of compound 1 in CD_3_OD, Figure S4: LC-MS analysis of compound 1, Figure S5: (**A**) The range of unigene length; (**B**) Unigene length distribution, Figure S6: Top-hit species similarity of assembled unigenes, Figure S7: The Gene Ontology (GO) pathway classification of assembled unigenes, Figure S8: The KEGG pathway classification of assembled unigenes, Figure S9: The visualization of differentially expressed genes (DEGs), Figure S10: WEGO enrichment comparison between annotated unigenes and AnLaeA upregulated genes in transformant OE::AnLaeA^11.19^, Figure S11: Plasmid maps, Table S1: Comparison between Az5LaeA and its orthologs, Table S2: Summary of RNA-seq statistics, Table S3: Statistics of the de novo assembly of RNA-seq, Table S4: Annotation of unigenes against different databases, Table S5: Primers used in this study, Table S6: List of plasmids used in this study, Table S7: List of LaeA regulated transcription factors, diorcinol-synthesis and velvet complex genes in *Aspergillus* sp. Z5
