# Supplementary material for: Heteroexpression of Aspergillus nidulans laeA in Marine-Derived Fungi Triggers Upregulation of Secondary Metabolite Biosynthetic Genes
Source: Mar Drugs. 2020 Dec 18;18(12):652. doi: 10.3390/md18120652 (PMC7766385; doi:10.3390/md18120652)
Supplement: Supplementary file 1 [file marinedrugs-18-00652-s001.pdf]

**Supplementary Material**

**Title: Heteroexpression of *Aspergillus nidulans laeA* in marine-derived fungi triggers upregulation of secondary metabolite biosynthetic genes**

Ishrat Khan[1], Wan-Lin Xie[1], Yu-Chao Yu[1], Huan Sheng[1], Yan-Xu[1], Jia-Qi Wang[1], Sanjit Chandra Debnath[1], Jin-Zhong Xu[1], Dao-Qiong Zheng[1], Wan-Jing Ding[1], Pin-Mei Wang[1,*]

**Affiliation**

1 Ocean College, Zhejiang University, Zhoushan, 316021, P. R. China

[*]Correspondence: wangpinmei@zju.edu.cn

```
Az5LaeA   68 NGRTYHGFRRGMYFLPCDEQEQDRLDIFHKLFTVARVSESLIYAPHPGNGRFLDLGCGTG
AnLaeA    77 NGRTYHGFRRGMYFLPCDEQEQDRLDIFHKLFTVARVSESLIYAPHPTNGRFLDLGCGTG
PzLaeA    82 NGRLYHGFRRGIYPLPCDEQEQDRLDIFHKLFTVARVSDGLIYAPHPSNGRVLDLGCGTG
PoLaeA    63 NNRLYHSYRKGTYMLPCDDEEQDRLDIFHKLFTVARVSDGLIYAPHPKNSRILDLGCGTG
MrLaeA    83 NGRIYHGFRRGIYFLPCDDLEQDRLDIFHKVITVARVSDALIYSPHPRNGRFLDLGCGTG
BsLaeA    84 NGRLYHGFRRGIYMLPCDEPEQDRLDIFHKLFTEARVSDGLIYAPHPENGRFLDLGCGTG
EgLaeA   116 NGRQYHGFRKGVYMYPCDDQEMDRLDIFHKLITEARVSDGLIYAPHPQNGRVLDLGCGTG
ReLaeA    90 NGRIYHGFKKGVYPFPCDEQEQDRLDIFHKVITEARIGDRLIYAPHSDDCRVLDLGCGTG

Az5LaeA  128 IWAIEVASKYPDAFVAGVDLAPIQPPNHPKNCEFYSPFDFEAPW-AMGEDSWDLIHLQMG
AnLaeA   137 IWAIEVANKYPDAFVAGVDLAPIQPPNHPKNCEFYAPFDFEAPW-AMGEDSWDLIHLQMG
PzLaeA   142 IWAIDVANKYPEAFVVGVDLAPIQPPNHPKNCDFYAPFDFESPW-AMGEESWDLIHMQMG
PoLaeA   123 IWSIEVANKYPDSFVVGVDLAPIQPQNHPSNCDFYAPFDFESPW-ALGEDSWDLIHMQMG
MrLaeA   143 IWAIDVAQKYPDAFVVGVDLSPIQPLNSPRNCDFYAPFDFESPW-ALGEDSWDLIHMQLG
BsLaeA   144 IWAIDIAHKYPNAHVVGVDLAPIQPSNRPKNCDFYAPWDFESHW-SLGEDSWDVVHMQMG
EgLaeA   176 IWAIDVAKKYPDAFVLGVDLSPIQPPNYPRNCDFYAPWDYDSPWPSLGEDSWDVIHLQMG
ReLaeA   150 IWAIDVANKYPKSFVVGVDLSPIQPKNIPKNCDFYAPWDFESPW-NLGENYWDLIHMQMG

Az5LaeA  187 CGSVMGWPNLYRRIFSHLRPGAWFEQVEIDFEPRCDDR--SLDGLALRHWYQYLKQATAE
AnLaeA   196 CGSVMGWPNLYRRIFAHLRPGAWFEQVEIDFEPRCDDR--SLDGTALRHWYDCLKQATAE
PzLaeA   201 SGSVASWPNLYRRIFAHLRPGAWFEQVEIDFEPRCDDR--SLDGLALRQWYQCLKQATEE
PoLaeA   182 SGSVASWPSLYRRIFSHLRPGAWFEQVEIDFEPRCDDR--SLSNLALRHWYTALKRATES
MrLaeA   202 CGSVVSWPSLYRRIFAHLRPGAWFEQVEIDFEPRCDDR--SLEGLALHHWYQCLKQATEE
BsLaeA   203 SGSVASWPSLYRRIFSHLRPGAWFEQVEIDFEPRCDDR--TLEGSALRSWYQWLKQATED
EgLaeA   236 CGSVTSWPSLYRRVFAHLRPGAWFEQVEIDFEPRCDDRSLALDNTALRRWYTLLKQATEQ
ReLaeA   209 AGSVTSWPSLYRRIFAHLRPGAWFEQVEIDFEPRCEGR--SLENTALSRWYQYLRQATEQ
```

**Figure S1.** Multiple sequence alignment of Az5LaeA with its orthologs by ClustalX2.1. Abbreviations and accession numbers: Az5LaeA, *Aspergillus* sp. Z5 LaeA, BK011996; AnLaeA, *Aspergillus nidulans* LaeA, XP_658411; PoLaeA, *Penicillium oxalicum* LaeA, EPS25650; PzLaeA, *Penicilliopsis zonata* LaeA, XP_022580130; MrLaeA, *Monascus ruber* LaeA, AIY63188; BsLaeA, *Byssochlamys spectabilis* LaeA, GAD98736; ReLaeA, *Rasamsonia emersonii* LaeA, XP_013330850; EgLaeA, *Elaphomyces granulatus* LaeA, OXV07344

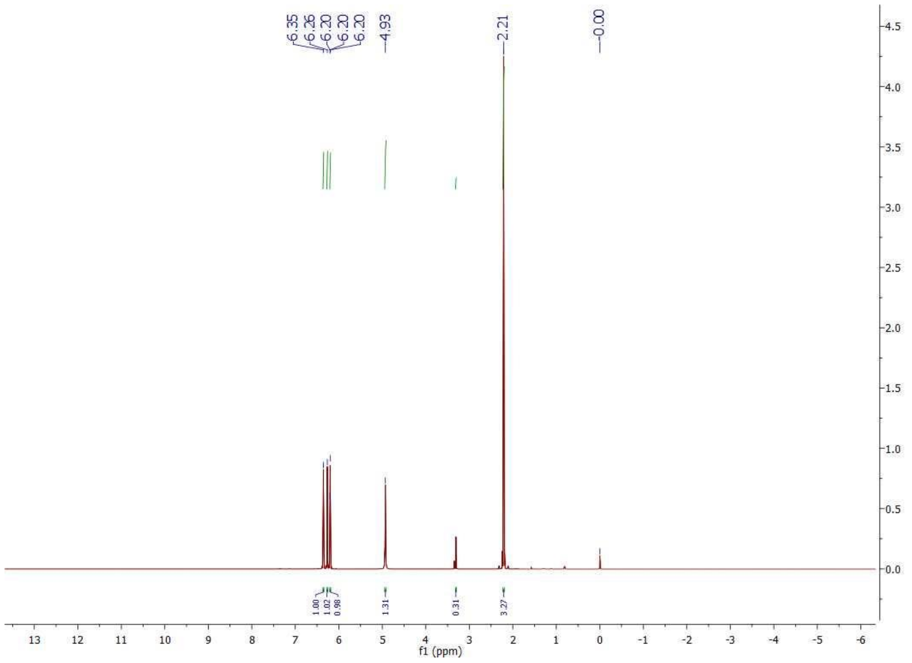


**Figure S2.** $^{1}$H NMR Spectrum of compound 1 in CD3OD

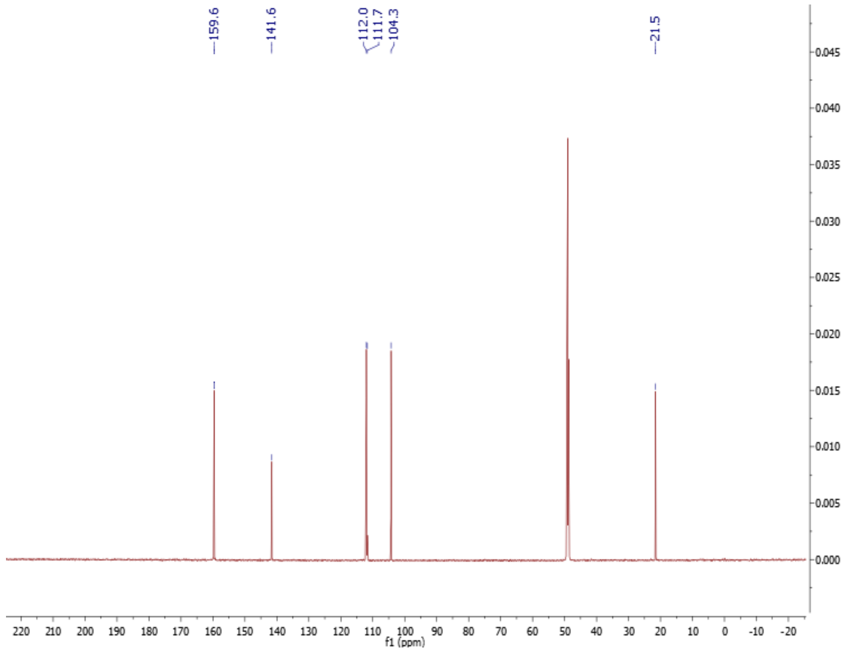


**Figure S3.** $^{13}C$ NMR Spectrum of compound 1 in CD3OD

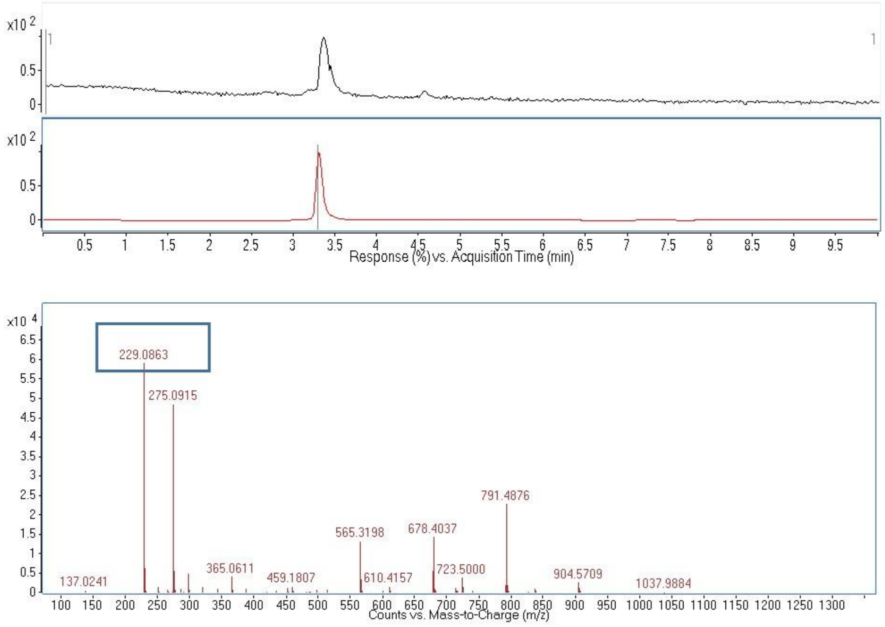


**Figure S4.** LC-MS analysis of compound 1

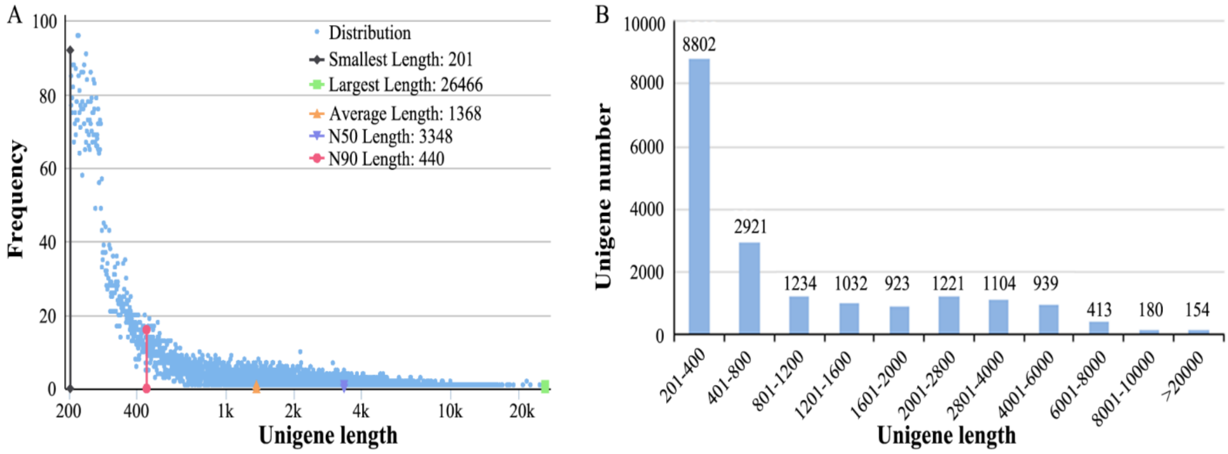


**Figure S5.** (**A**) The range of unigene length; (**B**) Unigene length distribution

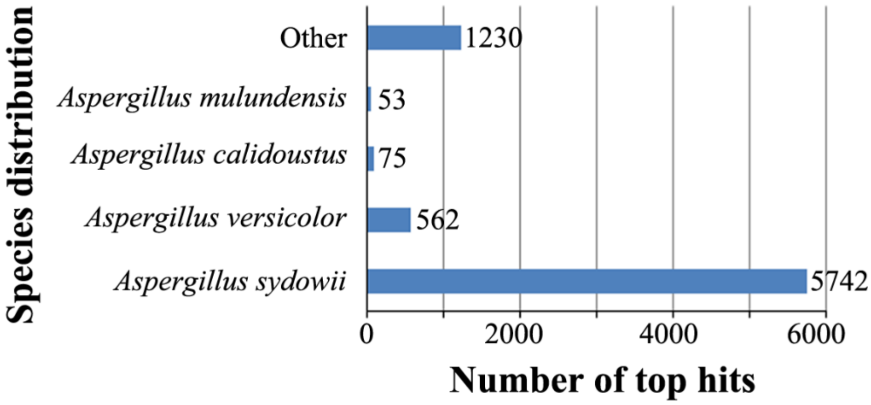


**Figure S6.** Top-hit species similarity of assembled unigenes

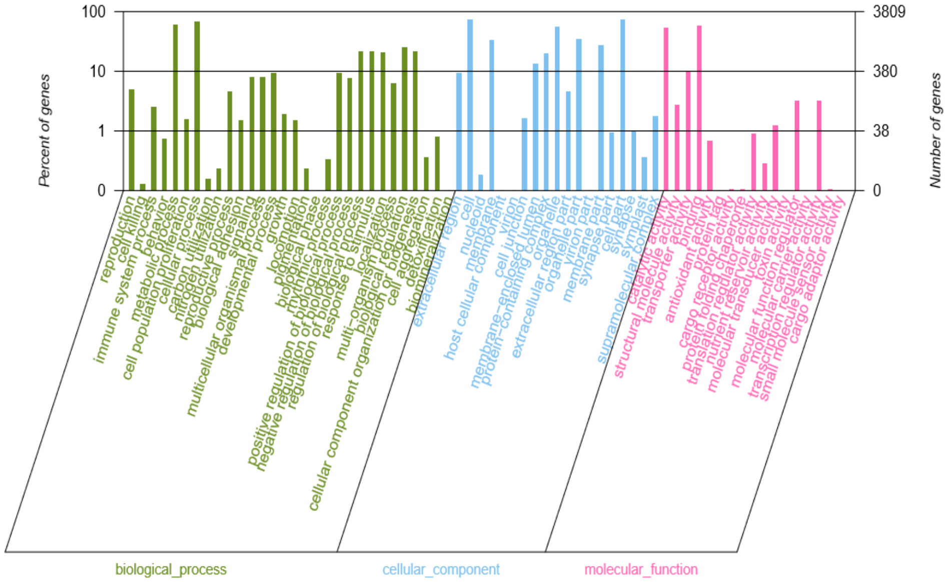


**Figure S7.** The Gene Ontology (GO) pathway classification of assembled unigenes

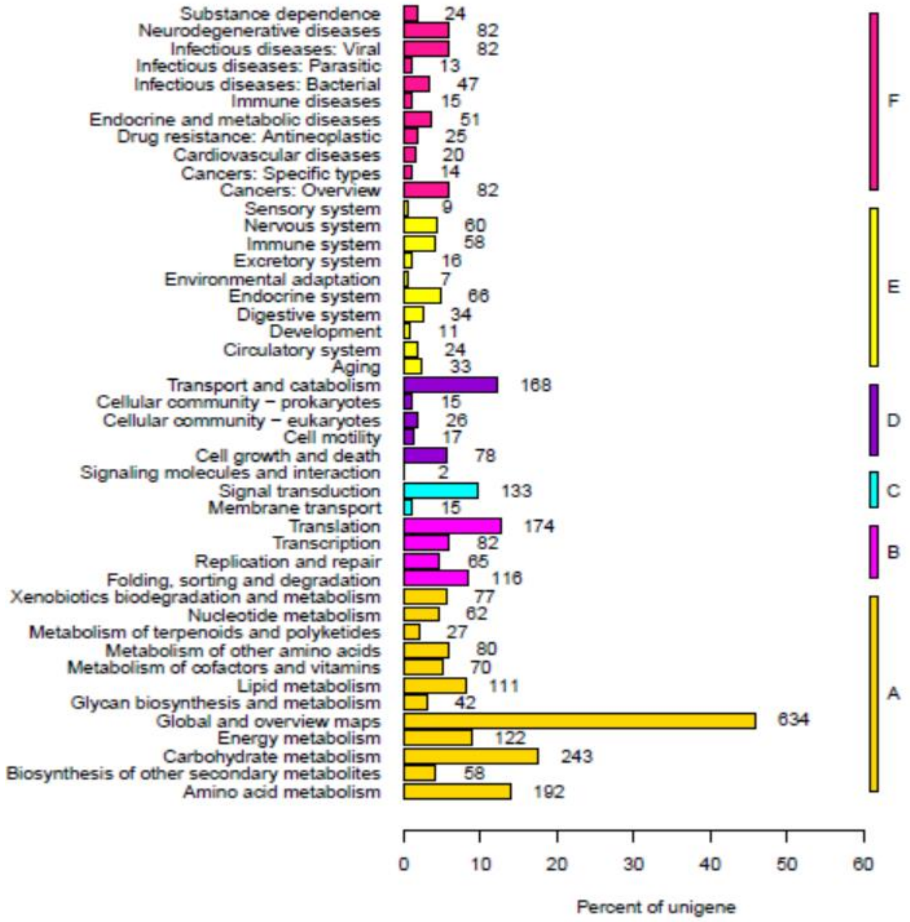


**Figure S8.** The KEGG pathway classification of assembled unigenes. (**A**) Metabolism; (**B**) Genetic Information Processing; (**C**) Environment Information Processing; (**D**) Cellular Process; (**E**) Organismal System; (**F**) Others

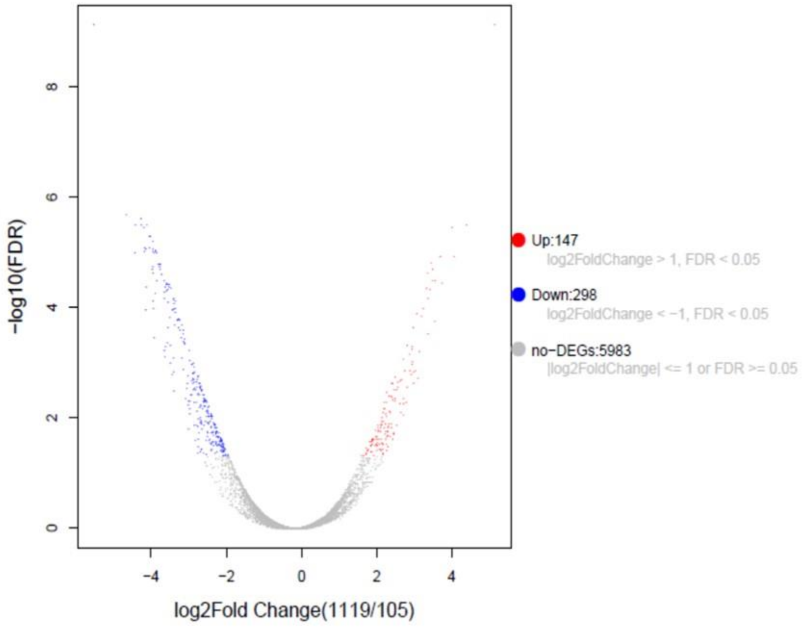


**Figure S9.** The visualization of differentially expressed genes (DEGs). Red dot: Significantly upregulated gene; Blue dot: Significantly down regulated gene; Grey dot: Not significantly differentially expressed gene.

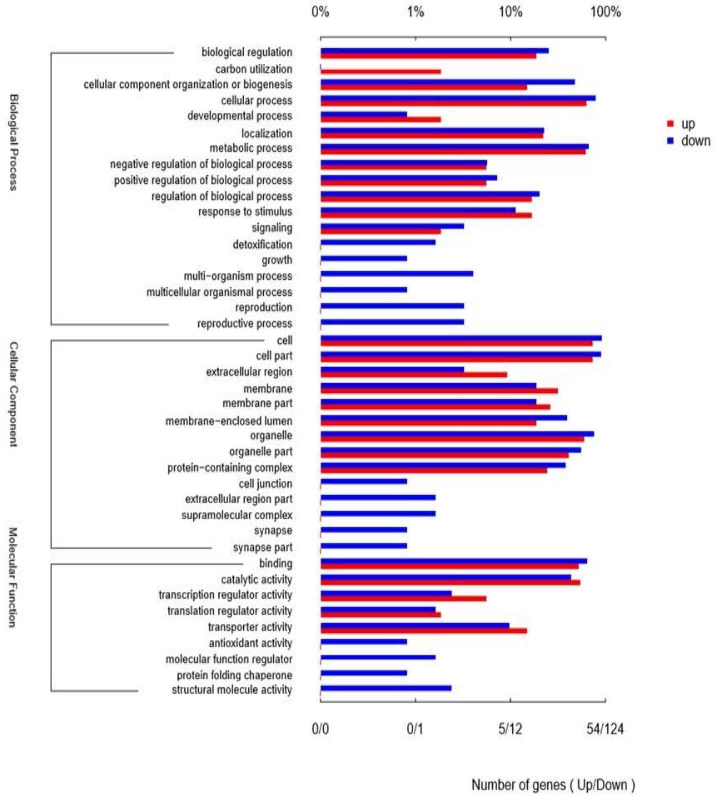


**Figure S10.** WEGO enrichment comparison between annotated unigenes and AnLaeA upregulated genes in transformant OE::AnLaeA[11.19].

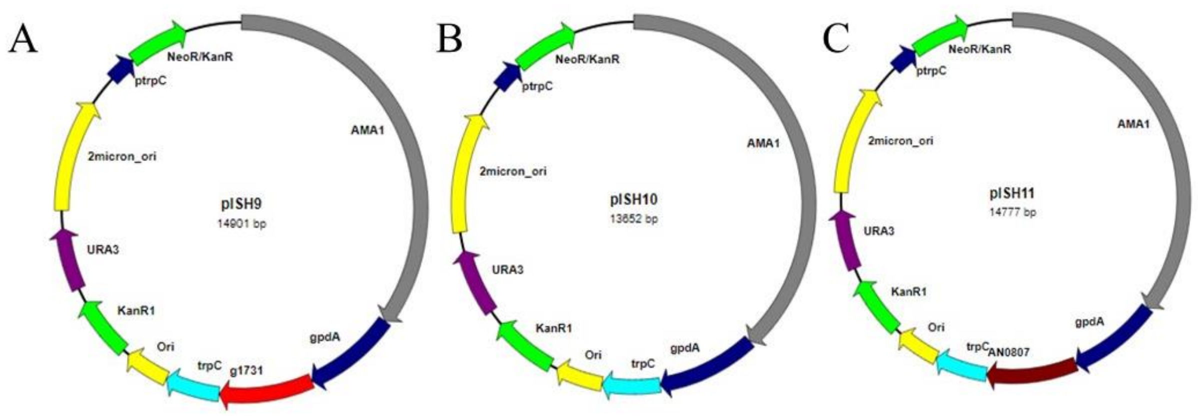


**Figure S11.** Plasmid maps. (**A**) *Aspergillus* sp. Z5 g1731.t1 *laeA* (Az5LaeA) overexpression plasmid; (**B**) Negative control plasmid; (**C**) *Aspergillus nidulans* AN0807 *laeA* (AnLaeA) overexpression plasmid

**Table S1.** Comparison between Az5LaeA and its orthologs

| **LaeA** | **Organism** | **Accession number** | **Length (aa)** | **Identity/coverage (%)** |
|---|---|---|---|---|
| Az5LaeA | *Aspergillus* sp. Z5 | BK011996 | 352 | - |
| AnLaeA | *Aspergillus nidulans* | XP_658411 | 361 | 86.07/95 |
| PoLaeA | *Penicillium oxalicum* | EPS25650 | 348 | 78.77/82 |
| PzLaeA | *Penicilliopsis zonata* | XP_022580130 | 367 | 73.84/99 |
| MrLaeA | *Monascus ruber* | AIY63188 | 368 | 71.10/93 |
| BsLaeA | *Byssochlamys spectabilis* | GAD98736 | 369 | 70.75/96 |
| ReLaeA | *Rasamsonia emersonii* | XP_013330850 | 377 | 66.67/89 |
| EgLaeA | *Elaphomyces granulatus* | OXV07344 | 404 | 60.15/99 |

**Table S2.** Summary of RNA-seq statistics

| | **nc10.5** | **OE::AnLaeA[11.19]** |
|---|---|---|
| Raw Reads | | |
| Total Reads | 50,033,398 | 48,146,046 |
| Total Bases (bp) | $7.51\times10^9$ | $7.22\times10^9$ |
| Clean Reads | | |
| Total Reads | 49,979,906 | 48,075,956 |
| Total Bases (bp) | $7.33\times10^9$ | $7.01\times10^9$ |
| Error % | 0.0266 | 0.0263 |
| Q20 % | 97.13 | 97.18 |
| Q30 % | 93 | 93.26 |
| GC % | 52.83 | 52.31 |

**Table S3.** Statistics of the *de novo* assembly of RNA-seq

| | Transcripts | Unigenes |
|---|---|---|
| Total number | 33,747 | 18,923 |
| Total Bases (bp) | 71,916,686 | 25,898,836 |
| GC % | 51.22 | 51.24 |
| Largest Length (bp) | 26,466 | 26,466 |
| Smallest Length (bp) | 200 | 201 |
| Average Length (bp) | 2131.05 | 1368.64 |
| N50 (bp) | 4614 | 3348 |
| N90 (bp) | 1052 | 440 |

**Table S4.** Annotation of unigenes against different databases

| Database / Type | Unigenes | Percentage % | Transcripts | Percentage % |
|---|---|---|---|---|
| Total number | 18,923 | - | 33,747 | - |
| NR | 7662 | 40.49 | 16,866 | 49.98 |
| Pfam | 3792 | 20.03 | 9575 | 28.37 |
| STRING | 5313 | 28.08 | 11,985 | 35.51 |
| SWISS-PROT | 3910 | 20.66 | 8309 | 24.62 |
| GO | 3809 | 20.12 | 8113 | 24.04 |
| KEGG | 2117 | 11.19 | 5133 | 15.21 |

**Table S5.** Primers used in this study

| Primers | Sequence *(5′-3′) | Purpose |
|---|---|---|
| ISH-Y LaeA E1F1 | ATGTTTGAGATGGGCCCGGTG | To clone coding sequence of AnLaeA gene |
| Y LaeA E2R2 | TTATCTTAATGGTTTCCTAGCCTGG | |
| ISH Z5 LaeA F1 | ATGTTTGGAAAGGGTCCGGTG | To clone Az5LaeA gene |
| ISH Z5 LaeA R1 | TTATCTCGACGGTTTTCGCG | |
| ISH gpdA F1 | *GAAAAACTTCGTGCTATCACAAAACAGTATACAAAAAATA*GCCGCACCTTCAGTGGACT | To clone gpdA promoter with AMA1 flanking region. |
| ISH gpdA R2 | TGTGATGTCTGCTCAAGCGGGGTAG | |

| Primers | Sequence *(5′-3′) | Purpose |
|---|---|---|
| ISH gpdA R1 | *GGGGTCGAGGTCCCACCGGACCCTTTCCAAACAT*TGTGATGTCTGCTCAAGCGGGGTAG | To clone gpdA promoter with Az5LaeA gene flanking region |
| ISH gpdA R3 | *GGAGACGAGTTCCCACCGGGCCCATCTCAAACAT*TGTGATGTCTGCTCAAGCGGGGTAG | To clone gpdA promoter with AnLaeA gene flanking region |
| ISH TtrpC F1 | *AGCTTGACTAACAGCTACCCCGCTTGAGCAGACATCACA*GGATCCACTTAACGTTACTG | To clone TtrpC terminator with gpdA flanking region |
| ISH TtrpC F2 | *ATACTGCACATATACCAGGCTAGGAAACCATTAAGATAA*GGATCCACTTAACGTTACTG | To clone TtrpC terminator with AnLaeA gene flanking region |
| ISH TtrpC F3 | *ATCCTACACATATACCAAGCGCGAAAACCGTCGAGATA*AGGATCCACTTAACGTTACTG | To clone TtrpC terminator with Az5LaeA gene and Ori flanking region |
| ISH TtrpC R1 | *ATTTTTGTGATGCTCGTCAGGGGGGCGGAGCCTATGGAAATA*GAAAGAAGGATTACCTC | |
| ISH-G418 F2 | TTTCCATAGGCTCCGCCCCCCTG | To clone vector backbone from pC-G418-YR vector with AMA1 flanking region |
| Y-G418 R1 | *CTTCGTGCTATCACAAAACAGTATACAAAAAATAAGCT*TAAACGCTCTTTTCTCTTAGG | |
| WJW-F | GCAAGGTGTTCAGAGACTA | To confirm AMA1 by diagnostic PCR |
| WJW-R | AGTGTTGGAAGTGATAGCAT | |
| ISH Neo/Kan F1 | ATGATTGAACAAGATGGATTGC | To confirm G418 gene by diagnostic PCR |
| ISH Neo/Kan R1 | TCAGAAGAACTCGTCAAGAAG | |
| ISH gpdA_ANLaeA F1 | ACGCAGAGAGAAGGGCTGAGTAATA | To confirm gpdA-AnLaeA gene fragment by diagnostic PCR |
| ISH gpdA_ANLaeA R1 | TGGTCGCCGGCTCTCGTTCA | |
| ISH gpdA_Az5LaeA F1 | GTAGGCAGCTTTGCCCCGTCT | To confirm gpdA-Az5LaeA gene fragment by diagnostic PCR |
| ISH gpdA_Az5LaeA R1 | TCGAAATCGAAGGGTGAATAGAAC | |
| Z5 VeA RT F1 | CGAACGACCACTTTACAATGGCA | To confirm Az5VeA gene expression by qPCR |
| Z5 VeA RT R1 | GCCCGCTTATAGGCCATCT | |
| Z5 VelB RT F1 | CCCTTCCAAGTGTTCTCTGCGA | |

| Primers | Sequence *(5′-3′) | Purpose |
|---|---|---|
| Z5 VelB RT R1 | CGTCCACGAGAGCCTTGAC | To confirm Az5VelB gene expression by qPCR |
| Z5aflR RTF1 | AGTATCGCAGGGCAGGCTG | To confirm Az5aflR1 gene |
| Z5aflR RTR1 | GAGTCCCGAGATCCACAGCT | expression by qPCR |
| Z5aflR RTF2 | AGAGGACGAGCGCAGGCTTA | To confirm Az5aflR2 gene |
| Z5aflR RTR2 | AACTGTTCCAGCGCCCCGT | expression by qPCR |
| Z5 sclR RTF1 | CTCTACTTCACGCTCTTCGAAA | To confirm Az5sclR gene |
| Z5 sclR RTR1 | ACGTACGCCGCCATCAAAA | expression by qPCR |
| Z5 GcnE RTF1 | TACAAGATCGACCCAGTAATGG | To confirm Az5GcnE gene |
| Z5 GcnE RTR1 | AACGGTGGCGACAGCTCGTT | expression by qPCR |
| Z5 mtfA RTF1 | AAGGCCTTCTCACGACCCT | To confirm Az5mtfA gene |
| Z5 mtfA RTR1 | TTCGTGGCGCTTCATATTGCT | expression by qPCR |
| Z5 sltA RTF1 | ATGGACTTCGCAGACTTCG | To confirm Az5sltA gene |
| Z5 sltA RTR1 | AGCAGAGTCATCTTCGAATTG | expression by qPCR |
| Z5 LlmF RTF1 | ATCCAAAACTGCACGAACTC | To confirm Az5LlmF gene |
| Z5 LlmF RTR1 | CACCTGGGCGCTTTCATACGA | expression by qPCR |
| Z5 mcrA RTF1 | AGGTCACACGCTTCTTTTACG | To confirm Az5mcrA gene |
| Z5 mcrA RTR1 | AAAAGATCCACGGGGCGAAG | expression by qPCR |
| Z5 OrsA RTF1 | CCGAGGATCTATCCGTTAA | To confirm Az5OrsA gene |
| Z5 OrsA RTR1 | CCCAGACCACAGCGACAT | expression by qPCR |
| Z5 OrsB RTF1 | GAGCAAATCAGCGACAGC | To confirm Az5OrsB gene |
| Z5 OrsB RTR1 | TTAGGGCACCGACGAATC | expression by qPCR |
| Z5 OrsC RTF1 | ATTTGGACGGTTTGGCAGG | To confirm Az5OrsC gene |
| Z5 OrsC RTR1 | ATGTCCGGACTGAAATCAAACA | expression by qPCR |
| Z5 OrsD RTF1 | ACCCGGCAGTCTTGGTGGTG | To confirm Az5OrsD gene |
| Z5 OrsD RTR1 | CAGGAAGCCTCTCAGCAATAC | expression by qPCR |
| Z5 OrsE RTF1 | TTAATGACATTGCGACTCCC | To confirm Az5OrsE gene |
| Z5 OrsE RTR1 | AGCGCCTCATCGACACTCTCA | expression by qPCR |
| Z5 OrsF RTF1 | AAACGTCAATGTAGGTTTCAGC | To confirm Az5OrsF gene |
| Z5 OrsF RTR1 | TCCCGATATCATCGCGCTCC | expression by qPCR |

* The italicized primer sequence is homologous 5′ flanking region to adjacent gene in vector construction

**Table S6.** List of plasmids used in this study

| Plasmid | Genotype | Reference |
|---|---|---|
| pISH1 | *amp oriC AnLaeAE1E2* | This study |
| pISH9 | *ama1 gpdA::Az5LaeA::trpC neoR/kanR* | This study |
| pISH10 | *ama1 gpdA trpC neoR/kanR* | This study |
| pISH11 | *ama1 gpdA::AnLaeA::trpC neoR/kanR* | This study |
| pC-G418 | *Ori neoR/kanR URA3 2micron* | http://www.addgene.org/61767/ |
| ANEp2 | *ama1 pyrG GlaPr lacA GlaTt pUC18* | http://fgsc.net/ |

**Table S7.** List of LaeA regulated transcription factors, diorcinol-synthesis and velvet complex genes in *Aspergillus* sp. Z5

| Genes | *A. nidulans*/ *A. oryzae* | *Aspergillus* sp. Z5 homologue (%Identity/%Positives/ Query coverage) | Function | Reference |
|---|---|---|---|---|
| Transcription factors | | | | |
| *aflR* | AN7820 | g378.t1(29/42/94) | Sterigmatocystin biosynthesis regulator | [1] |
| *gcnE* | AN3621 | g3373.t1 (94/96/99) | Acetyltransferase in SAGA complex | [2] |
| *llmF* | AN6749 | g9402.t1 (76/83/96) | LaeA-like methyltransferase | [3] |
| *mcrA* | AN8694 | g6964.t1 (68/74/98) | Master regulator of secondary metabolism in *Aspergillus* | [4] |
| *mtfA* | AN8741 | g2250.t1 (78/85/99) | Putative C2H2 transcription factor involve in regulation of secondary metabolism | [5] |
| *sclR* | AO090011000215 | g6252.t1 (65/70/25) | Hyphal morphology, asexual conidiospore formation and sclerotial production | [6] |
| *sltA* | AN2919 | g7633.t1 (78/86/99) | C2H2 transcription factor involve in cation homeostasis | [7] |

| Genes | *A. nidulans*/ *A. oryzae* | *Aspergillus* sp. Z5 homologue (%Identity/%Positives/ Query coverage) | Function | Reference |
|---|---|---|---|---|
| Diorcinol biosynthesis | | | | |
| *orsA* | AN7909 | g2173.t1 (77/85/100) | Polyketide synthase; F9775 secondary metabolite gene cluster | [8] |
| *orsB* | AN7911 | g2171.t1 (85/92/98) | Putative amidohydrolase; F9775 secondary metabolite gene cluster | [8] |
| *orsC* | AN7912 | g2170.t1 (79/87/98) | Putative tyrosinase; F9775 secondary metabolite gene cluster | [8] |
| *orsD* | AN7913 | g2169.t1 (64/75/98) | F9775 secondary metabolite gene cluster | [9] |
| *orsE* | AN7914 | g2168.t1 (87/93/99) | Putative alcohol dehydrogenase; F9775 secondary metabolite gene cluster | [9] |
| *orsF* | AN7910 | g2172.t1 (78/88/93) | Hypothetical protein | [10] |
| Velvet complex | | | | |
| *veA* | AN1052 | g4963.t1 (73/79/99) | Coordinates development and secondary metabolism in response to light | [11] |
| *velB* | AN0363 | g142.t1 (80/87/95) | Coordinates development and secondary metabolism in response to light | [11] |

**References**


1. Bok, J.W.; Keller, N.P. LaeA, a regulator of secondary metabolism in *Aspergillus* spp. *Eukaryot. cell* **2004**, *3*, 527-535, doi:10.1128/ec.3.2.527-535.2004.

2. Georgakopoulos, P.; Lockington, R.A.; Kelly, J.M. The Spt-Ada-Gcn5 Acetyltransferase (SAGA) complex in *Aspergillus nidulans*. *PloS one* **2013**, *8*, e65221-e65221, doi:10.1371/journal.pone.0065221.
3. Palmer, J.M.; Theisen, J.M.; Duran, R.M.; Grayburn, W.S.; Calvo, A.M.; Keller, N.P. Secondary metabolism and development is mediated by LlmF control of VeA subcellular localization in *Aspergillus nidulans*. *PLoS Genet.* **2013**, *9*, e1003193, doi:10.1371/journal.pgen.1003193.
4. Oakley, C.E.; Ahuja, M.; Sun, W.-W.; Entwistle, R.; Akashi, T.; Yaegashi, J.; Guo, C.-J.; Cerqueira, G.C.; Russo Wortman, J.; Wang, C.C.C., et al. Discovery of McrA, a master regulator of *Aspergillus* secondary metabolism. *Mol. Microbiol.* **2017**, *103*, 347-365, doi:10.1111/mmi.13562.
5. Ramamoorthy, V.; Dhingra, S.; Kincaid, A.; Shantappa, S.; Feng, X.; Calvo, A.M. The putative C2H2 transcription factor MtfA is a novel regulator of secondary metabolism and morphogenesis in *Aspergillus nidulans*. *PloS one* **2013**, *8*, e74122-e74122, doi:10.1371/journal.pone.0074122.
6. Jin, F.J.; Takahashi, T.; Matsushima, K.-i.; Hara, S.; Shinohara, Y.; Maruyama, J.-i.; Kitamoto, K.; Koyama, Y. SclR, a basic helix-loop-helix transcription factor, regulates hyphal morphology and promotes sclerotial formation in *Aspergillus oryzae*. *Eukaryot. cell* **2011**, *10*, 945-955, doi:10.1128/EC.00013-11.
7. Shantappa, S.; Dhingra, S.; Hernández-Ortiz, P.; Espeso, E.A.; Calvo, A.M. Role of the zinc finger transcription factor SltA in morphogenesis and sterigmatocystin biosynthesis in the fungus *Aspergillus nidulans*. *PloS one* **2013**, *8*, e68492-e68492, doi:10.1371/journal.pone.0068492.
8. Sanchez, J.F.; Chiang, Y.-M.; Szewczyk, E.; Davidson, A.D.; Ahuja, M.; Elizabeth Oakley, C.; Woo Bok, J.; Keller, N.; Oakley, B.R.; Wang, C.C.C. Molecular genetic analysis of the orsellinic acid/F9775 gene cluster of *Aspergillus nidulans*. *Mol. Biosyst.* **2010**, *6*, 587-593, doi:10.1039/b904541d.
9. Bok, J.W.; Chiang, Y.-M.; Szewczyk, E.; Reyes-Dominguez, Y.; Davidson, A.D.; Sanchez, J.F.; Lo, H.-C.; Watanabe, K.; Strauss, J.; Oakley, B.R., et al. Chromatin-level regulation of biosynthetic gene clusters. *Nat. Chem. Biol.* **2009**, *5*, 462-464, doi:10.1038/nchembio.177.
10. Galagan, J.E.; Calvo, S.E.; Cuomo, C.; Ma, L.J.; Wortman, J.R.; Batzoglou, S.; Lee, S.I.; Baştürkmen, M.; Spevak, C.C.; Clutterbuck, J., et al. Sequencing of *Aspergillus nidulans* and comparative analysis with *A. fumigatus* and *A. oryzae*. *Nature* **2005**, *438*, 1105-1115, doi:10.1038/nature04341.
11. Bayram, O.; Krappmann, S.; Ni, M.; Bok, J.W.; Helmstaedt, K.; Valerius, O.; Braus-Stromeyer, S.; Kwon, N.J.; Keller, N.P.; Yu, J.H., et al. VelB/VeA/LaeA complex coordinates light signal

with fungal development and secondary metabolism. *Science* **2008**, *320*, 1504-1506, doi:10.1126/science.1155888.
